# A Fast Time-Adaptive Data Association Method for Multi-Target Tracking with Discontinuous Sparse LEO Satellite Observations

**DOI:** 10.3390/s26154842

**Published:** 2026-08-01

**Authors:** Dandan Wang, Zhi Yang, Xinli Zhu, Jinhao Gao, Yasheng Zhang

**Affiliations:** 1 Department of Aerospace Engineering and Technology, Space Engineering University, Beijing 101416, China; hgd_wdd@hgd.edu.cn (D.W.); zhuxinli2021@hgd.edu.cn (X.Z.); goldseu01@163.com (J.G.); 2 DFH Satellite Co., Ltd., Beijing 100094, China; young_zhi@163.com

**Keywords:** multi-target tracking, data association, discontinuous sparse observations, time-interval adaptive gating, annealing-based clustering

## Abstract

**Highlights:**

**What are the main findings?**

- The time-interval adaptive gating (TIAG) mechanism preserves the gating capture probability under arbitrary revisit intervals, compensating for covariance mismatch induced by discontinuous sparse observations.
- A simulated annealing-based progressive clustering strategy recursively decomposes the global exponential association graph into independent subgraphs of manageable sizes, significantly reducing the computational complexity of JPDA.

**What are the implications of the main findings?**

- The proposed method enables continuous and robust multi-target tracking for LEO satellite constellations under non-uniform and intermittent observations, with improved tracking accuracy and track completeness.
- With real-time computational efficiency and a hybrid safety-degradation mechanism, the method is well-suited for onboard or edge deployment in maritime surveillance and remote sensing applications.

**Abstract:**

In low-Earth-orbit (LEO) satellite constellation remote sensing for surface maritime target detection, the inherent characteristics of discontinuous detection epochs, non-uniform temporal intervals, and clutter contamination invariably cause conventional data association algorithms to suffer from validation gate degradation, covariance divergence, and combinatorial explosion. To circumvent these limitations, this paper proposes a multi-target, time-adaptive fast association method tailored for discontinuous sparse observations. Within the joint probabilistic data association (JPDA) framework, the proposed method analyzes the mismatch between the Kalman filter prediction covariance and the actual error under discontinuous observations. A time-interval adaptive gating mechanism maintains the gate detection probability across arbitrary revisit intervals. Secondly, to resolve the massive connected cluster problem triggered by the densification of the validation matrix, a progressive clustering strategy inspired by simulated annealing is designed, which recursively decomposes the global, exponentially scaling association graph into independent subgraphs of manageable sizes. Building upon this, a depth-first search (DFS) heap pruning technique is integrated with the Hungarian hard assignment algorithm as a safety-degradation mechanism to safeguard numerical robustness in extreme scenarios. Comparative experiments demonstrate that the proposed method significantly enhances both tracking accuracy and track completeness across various constellation coverage characteristics and maritime clutter intensities. Furthermore, its execution efficiency satisfies real-time simulation requirements, effectively supporting engineering application for surface maritime target detection.

## 1. Introduction

With the exponential scaling of low-Earth-orbit (LEO) satellite constellations, space-based wide-area remote sensing systems have demonstrated unprecedented potential in domains such as Earth-surface maritime traffic management [1], illegal fishing monitoring [2], and the safeguarding of maritime rights and interests [3]. Compared with ground-based radars and airborne platforms, LEO satellite constellations offer expansive coverage, short revisitation cycles, and independence from geopolitical boundaries, making them suitable for global wide-area detection. Space-based remote sensing systems typically collect noise-contaminated observation data through periodic detection, subsequently utilizing data association algorithms to synthesize continuous target trajectories. However, governed by orbital revisitation constraints, the observation data acquired by space-based detection payloads typically exhibit distinctive characteristics such as discontinuity, large temporal gaps, sparse spatial distribution, and intense measurement noise. When superimposed with environmental factors like complex maritime weather, sea clutter, and electromagnetic interference, the problems of data missingness and distortion are further exacerbated. This leads to severe trajectory confusion, rendering conventional data association algorithms ill-suited for such intricate application scenarios.

Currently, mainstream multi-target data association algorithms confront severe bottlenecks in discontinuous and sparse observation scenarios. The Nearest Neighbor (NN) [4] and Global Nearest Neighbor (GNN) [5] algorithms heavily rely on spatial proximity metrics for data assignment; consequently, they are highly susceptible to false associations and track fragmentation under sparse distributions. Multiple Hypothesis Tracking (MHT) maintains alternative association hypotheses through multi-branch tree structures; however, its substantial memory overhead and prohibitive inference latency impede its deployment on resource-constrained platforms, such as on-board or edge devices, failing to satisfy real-time constraints [6]. To alleviate the computational burden of MHT, subsequent studies have proposed various optimization strategies, such as engineering implementations leveraging pruning and merging techniques [7,8]; nevertheless, balancing accuracy and efficiency under conditions of prolonged missed detections and intense clutter remains a daunting challenge. The Joint Probabilistic Data Association (JPDA) algorithm computes joint posterior probabilities between measurements and targets and handles multi-target association in dense clutter [9]. Regrettably, the standard JPDA framework is predicated on the idealized assumptions of uniform sampling and a constant state transition matrix, making it difficult to directly adapt to actual space-based detection environments where observation intervals fluctuate dynamically and drastically. Furthermore, the computational complexity of the JPDA algorithm scales exponentially with the number of targets and measurements, severely restricting its utility in real-time systems.

To mitigate the computational complexity of the JPDA algorithm, a classic and effective paradigm involves partitioning targets without overlapping validation gates into independent calculations via clustering or pooling techniques, thereby grouping different measurements and gates separately [10]. Regarding computational efficiency and complexity reduction, Rezatofighi et al. [11] transformed the assignment score computation of JPDA into a sequence of Integer Linear Programming (ILP) problems, employing the m-best algorithm to approximate the joint scores. Liu et al. [12] integrated the NN algorithm with JPDA to propose a k-nearest neighbor joint probabilistic data association algorithm, which reduces the number of joint events and improves data association efficiency. Lee et al. [13] introduced a Markov Chain Joint Integrated Probabilistic Data Association (MCJIPDA) algorithm, which approximates multi-target data association probabilities by generating a Markov chain of target clusters, significantly lowering computational complexity. Qu et al. [14] proposed a reinforcement learning-based JPDA (RL-JPDA) that dynamically tunes policy parameters in conjunction with target motion characteristics and measurement distributions to enhance JPDA accuracy. Nazari et al. [15] proposed a fuzzy data association method based on density clustering, which utilizes density clustering to directly extract validated measurements for each target, thereby bypassing traditional validation gate design. By selecting key measurements with high membership degrees for state updates, this method significantly reduces computational complexity while maintaining tracking accuracy. For high-resolution maritime radar data, Fowdur et al. [16] utilized DBSCAN clustering to preprocess point clouds, decomposing the multi-target association problem into multiple independent subproblems, which effectively reduced the joint event scale of JPDA and boosted the overall efficiency of extended target tracking. Despite these advancements, existing methods still struggle with covariance mismatch under discontinuous observations, combinatorial explosion from massive connected clusters, and numerical instability under intense clutter.

Beyond data association algorithms, recent advances in nonlinear uncertainty propagation and high-order Taylor series methods offer complementary perspectives for handling the covariance mismatch problem under nonlinear dynamics. Boone and McMahon [17] proposed the directional state transition tensor (DSTT) method, which captures dominant nonlinear effects in orbital dynamics by aligning the state transition tensors (STTs) along the most sensitive directions, significantly reducing the computational and storage requirements of high-order STTs. Zhou et al. [18] further extended this concept to a time-varying DSTT (TDSTT), which integrates eigenvalue–eigenvector pairs and high-order Taylor series expansions simultaneously, enabling uncertainty propagation analysis at arbitrary epochs along the trajectory. These methods provide efficient approximations of nonlinear covariance evolution and have been successfully applied to orbit uncertainty propagation in highly nonlinear three-body systems. In parallel, differential algebra (DA) techniques have been developed for multi-target tracking and data association of too-short arcs. Pirovano et al. [19] introduced a DA-enabled admissible region (AR) sampling method that propagates uncertainties as Taylor polynomials and performs correlation via initial value and boundary value formulations with range intersection. These DA-based methods demonstrate how analytical uncertainty representation can be exploited for robust short-arc association, which shares conceptual similarities with our adaptive gating framework in dealing with sparse and uncertain observations. Collectively, these works highlight the importance of capturing dominant uncertainty directions and nonlinear effects, which aligns with the motivation of our TIAG mechanism to compensate for covariance mismatch under discontinuous observations.

To confront the aforementioned challenges, this paper proposes a multi-target, time-interval adaptive fast association method adapted for discontinuous sparse observations. Building upon the classic JPDA framework, the proposed method incorporates a variable-interval Kalman filter prediction mechanism and designs an integrated tracker comprising time-interval adaptive gating, annealing-based clustering (ABC), heuristic pruning joint event search, and a hybrid safety-degradation mechanism. This architecture enables continuous tracking of discontinuous and non-uniform observation data. The primary contributions of this paper are summarized as follows:

(1) Time-interval Adaptive Gating (TIAG) Mechanism: An analytical mapping between satellite revisit intervals and gating thresholds is derived, with an adaptive threshold tuning strategy that compensates for validation gate degradation under discontinuous observations for Earth-surface moving targets.
(2) Annealing-Based Clustering Algorithm: A progressive graph partitioning algorithm based on an annealing strategy recursively decomposes the dense association graph into manageable subgraphs, preserving association completeness and bounding computational complexity in surface-target tracking scenarios.
(3) Hybrid Safety-Degradation Mechanism: A hybrid degradation strategy is developed by synthesizing a K-best DFS heap pruning algorithm with the Hungarian hard assignment algorithm. When the cluster size exceeds a pre-specified safety threshold, the system automatically switches to hard-assignment mode, safeguarding numerical robustness in extreme scenarios for discontinuous surface observations.

## 2. Time-Interval Adaptive Gating Mechanism

Discontinuous sparse observation refers to a mode where, due to long satellite revisit cycles and environmental occlusions, measurements of the same target exhibit non-uniform temporal sampling, low spatial density, and intermittent interruptions. Under this mode, targets fail to yield effective measurements during every satellite overflight of the mission area, frequently resulting in consecutive multi-frame missed detections. Most conventional tracking algorithms are predicated on the assumptions of uniform sampling and dense measurements; consequently, in non-ideal scenarios, they face critical challenges such as validation gate failure and state covariance divergence, making it difficult to guarantee tracking stability and accuracy. This paper proposes a time-interval adaptive gating (TIAG) mechanism that compensates for covariance mismatch under discontinuous observations, improving track association stability.

### 2.1. Target Motion Model Under Discontinuous Sparse Observations

A mathematical model for discontinuous sparse space-based detection is established, which can be leveraged to acquire space-based observation data via STK (v10.1) simulations [20]. The moment a target enters the satellite’s FOV marks the start time, and its departure marks the end time. The space-based detection system registers the detection epoch of a target at the start time. Consequently, the constellation’s tracking of moving maritime targets exhibits typically non-uniform detection characteristics.

Although spaceborne payloads inherently operate within a three-dimensional geometric space, the maritime moving targets evaluated in this study are strictly surface vessels. Consequently, their vertical dynamics—such as heave, pitch, and roll—are negligible compared to their horizontal displacements. A local tangent plane approximation is adopted within the operational domain spanning approximately $1^{\circ }\times 1^{\circ }$ in latitude and longitude. The raw satellite measurements, originally resolved in the Earth-Centered Earth-Fixed (ECEF) frame, are transformed into the local East–North–Up (ENU) coordinate system, where only the east and north (horizontal) components are retained to form a two-dimensional state vector [21]. This dimensional reduction reduces the computational complexity of the data association algorithm without compromising tracking accuracy under the evaluated scenarios. Furthermore, the Time-interval Adaptive Gating (TIAG) and annealing clustering methods developed in this work are mathematically generalizable across state dimensions, permitting extension to three-dimensional state spaces—such as incorporating altitude data or directly utilizing geodetic coordinates—in future research.

The maritime target is modeled as executing Constant Velocity (CV) rectilinear motion within a two-dimensional plane. For open-ocean ships, this assumption is physically reasonable as they typically maintain near-constant speed along straight-line routes to minimize fuel consumption, consistent with standard rhumb-line navigation practice. This CV model is widely adopted as a baseline in maritime tracking [22], particularly for gating design where the primary objective is to define a search region rather than precise motion prediction. The simulation scenarios use rectilinear motion, consistent with the CV assumption and typical merchant ship navigation.

The state vector of target j at epoch $t_{k}$ is defined as $x_{j,k}=[x,y,v_{x},v_{y}{]}^{⊤}\in R^{4}$, where $\left(x,y\right)$ denotes the position coordinates, and $\left(v_{x},v_{y}\right)$ represents the velocity components. The state transition matrix F(Δt) and the process noise covariance matrix $Q\left(\Delta t\right)$ are both functions of the time interval Δt [23]:

(1) $$F(\Delta t)=\left[\begin{array}{cccc} 1 & 0 & \Delta t & 0\\ 0 & 1 & 0 & \Delta t\\ 0 & 0 & 1 & 0\\ 0 & 0 & 0 & 1 \end{array}\right],\;Q\left(\Delta t\right)=q\left[\begin{array}{cccc} \frac{\Delta t^{3}}{3} & 0 & \frac{\Delta t^{2}}{2} & 0\\ 0 & \frac{\Delta t^{3}}{3} & 0 & \frac{\Delta t^{2}}{2}\\ \frac{\Delta t^{2}}{2} & 0 & \Delta t & 0\\ 0 & \frac{\Delta t^{2}}{2} & 0 & \Delta t \end{array}\right],$$

where q signifies the process noise intensity coefficient. The measurement model is formulated as a linear observation:

(2) $$z_{k}=Hx_{k}+v_{k},$$

where the observation matrix is $H=[I_{2},0_{2}]$, and the measurement noise follows a Gaussian distribution $v_{k}∼N(0,R)$, with $R=\sigma_{R}^{2}I_{2}$.

For target j, given its state estimate ${\hat{x}}_{j,k-1}$ and error covariance $P_{j,k-1}$ at epoch k−1, the prediction equations to epoch k are expressed as:

(3) $${\hat{x}}_{j,k|k-1}=F(\Delta t_{k}){\hat{x}}_{j,k-1},$$

(4) $$P_{j,k|k-1}=F(\Delta t_{k})P_{j,k-1}F(\Delta t_{k}{)}^{⊤}+Q(\Delta t_{k}).$$

The innovation vector and its theoretical covariance are respectively given by, where the superscript $\left(i\right)$ denotes the satellite measurement index and the subscript *j* denotes the target index:

(5) $${\tilde{z}}_{ij}=z_{k}^{\left(i\right)}-H{\hat{x}}_{j,k|k-1},S_{ij}=HP_{j,k|k-1}H^{⊤}+R.$$

### 2.2. Conventional Joint Data Association Gating Methods

Let ${\hat{x}}_{j,k|k-1}$ denote the predicted state of target j at epoch k, and $P_{j,k|k-1}$ represent its predicted error covariance. For the *i*-th satellite measurement $z_{k}^{\left(i\right)}$, with the innovation vector ${\tilde{z}}_{ij}$ and the innovation covariance $S_{ij}$ defined as in Equation (5), the Mahalanobis distance test statistic is given by:

(6) $$d_{ij}^{2}={\tilde{z}}_{ij}^{⊤}S_{ij}^{-1}{\tilde{z}}_{ij}\leq \gamma ,$$

where the threshold γ is typically chosen as the quantile of a chi-squared distribution with $n_{z}$ (measurement dimension) degrees of freedom. For instance, when $n_{z}=2$ and the confidence level is 99%, the baseline threshold is $\gamma_{0}=\chi_{2}^{2}(0.99)\approx 9.21$. The theoretical premise of this fixed threshold is a constant sampling interval Δt, under which $P_{j,k|k-1}$ smoothly converges over successive frames, forcing the Mahalanobis distance to strictly adhere to a $\chi_{n_{z}}^{2}$ distribution [24].

### 2.3. Covariance Mismatch Mechanism Analysis Under Discontinuous Observations

In practical target tracking systems, when the sensor sampling interval Δt is large, the pre-specified target motion model fails to accurately capture the true trajectory of the target. During actual movement, targets are invariably subject to unknown environmental perturbations, such as slight maneuvers, marine currents, or aerodynamic fluctuations. Let the true acceleration a be a two-dimensional random vector satisfying E[a]=0, and possessing a covariance of $E[aa^{⊤}]=\sigma_{a}^{2}I_{2}$, where $\sigma_{a}^{2}$ denotes the variance of the environmental perturbation. These unmodeled environmental disturbances introduce supplementary uncertainties into the predicted positions and velocities, systematically underestimating the state covariance matrix computed by the filter.

#### 2.3.1. Prediction Error Induced by Acceleration

Consider a two-dimensional discrete-time system whose state vector is defined as $x={\left[x,y,\dot{x},\dot{y}\right]}^{T}$. The true state evolution equation incorporating the unknown acceleration is formulated as:

(7) $$x_{k}=F(\Delta t)x_{k-1}+\Gamma (\Delta t)a_{k-1}+w_{k-1},$$

where F(Δt) is the state transition matrix, and $\Gamma \left(\Delta t\right)$ is the acceleration input matrix:

(8) $$F\left(\Delta t\right)=\left[\begin{array}{cc} I_{2} & \Delta tI_{2}\\ 0_{2} & I_{2} \end{array}\right],\;\;\Gamma \left(\Delta t\right)=\left[\begin{array}{c} \frac{1}{2}\Delta t^{2}I_{2}\\ \Delta tI_{2} \end{array}\right].$$

The standard Kalman filter assumes the target undergoes constant velocity motion, i.e., a=0. yielding the predicted state ${\hat{x}}_{k|k-1}^{\mathrm{filter}}=F(\Delta t){\hat{x}}_{k-1}$. The error between the true state and the filter’s prediction is given by:

(9) $${\tilde{x}}_{k|k-1}=x_{k}-{\hat{x}}_{k|k-1}^{\mathrm{filter}}=F\left(\Delta t\right)\left(x_{k-1}-{\hat{x}}_{k-1}\right)+\Gamma \left(\Delta t\right)a+w_{k-1}.$$

The second term in the equation denotes the error induced by acceleration perturbations, which arises from external disturbances and cannot be modeled precisely.

#### 2.3.2. Additional Covariance Induced by Acceleration

It is important to distinguish between the process noise covariance Q(Δt) in Equation (4), which is a design parameter specified by the filter to account for assumed acceleration uncertainty, and the additional covariance ΔP(Δt) derived below, which quantifies the actual mismatch between the true acceleration and the zero-acceleration assumption of the CV model. In a well-tuned filter, Q(Δt) can be chosen to cover such mismatches. However, the purpose of the following derivation is not to propose a new covariance term for the filter implementation, but rather to analytically expose the mechanism by which unmodeled acceleration—in the presence of large and varying Δt—causes the true innovation covariance to deviate from the filter-calculated nominal value. This deviation is the root cause of the fixed-gating failure analyzed in Section 2.3.3.

The additional uncertainty induced by the unmodeled acceleration relative to the nominal CV assumption is:

(10) $$P_{k|k-1}^{\mathrm{true}}=P_{k|k-1}^{\mathrm{filter}}+\Gamma (\Delta t)\;E[aa^{⊤}]\;\Gamma (\Delta t{)}^{⊤}.$$

This term represents the covariance contribution from the unknown acceleration a that is not accounted for by the filter’s assumed zero-acceleration model. Substituting $E[aa^{T}]=\sigma_{a}^{2}I_{2}$ and computing $\Gamma \Gamma^{T}$ yields:

(11) $$\Gamma (\Delta t)\Gamma (\Delta t{)}^{⊤}=\left[\begin{array}{cc} \frac{\Delta t^{4}}{4}I_{2} & \frac{\Delta t^{3}}{2}I_{2}\\ \frac{\Delta t^{3}}{2}I_{2} & \Delta t^{2}I_{2} \end{array}\right].$$

Therefore,

(12) $$\Delta P(\Delta t)=\sigma_{a}^{2}\Gamma (\Delta t)\Gamma (\Delta t{)}^{⊤}.$$

From the derivation above, it is clear that the additional uncertainty ΔP(Δt) and the process noise covariance Q(Δt) serve different roles. While Q(Δt) is a filter design parameter that can be adjusted to absorb unmodeled dynamics, ΔP(Δt) quantifies the actual mismatch between the assumed and true motion. The key insight for the TIAG mechanism is that the mismatch grows rapidly with Δt as $\Delta t^{4}$ (see Equation (15)), which cannot be fully compensated by a fixed Q(Δt) when the revisit interval varies significantly. This scaling mismatch—rather than the presence of acceleration per se—is what drives the failure of fixed gating thresholds under discontinuous observations.

#### 2.3.3. Failure Mechanism of Fixed Gating Thresholds

During the measurement update stage, the nominal innovation covariance computed by the filter is:

(13) $$S^{\mathrm{filter}}=HP_{k|k-1}^{\mathrm{filter}}H^{⊤}+R.$$

When augmented with the acceleration perturbation, the true innovation covariance becomes:

(14) $$S^{\mathrm{true}}=S^{\mathrm{filter}}+H\Delta P(\Delta t)H^{⊤}.$$

Given that the observation matrix is **H** $={\left[I_{2},0_{2}\right]}^{T}$, direct substitution yields:

(15) $$H\Delta P(\Delta t)H^{⊤}=\sigma_{a}^{2}\frac{\Delta t^{4}}{4}I_{2}.$$

Thus, we obtain:

(16) $$S^{\mathrm{true}}=S^{\mathrm{filter}}+\sigma_{a}^{2}\frac{\Delta t^{4}}{4}I_{2}.$$

As Δt scales up, the true measurement prediction covariance significantly exceeds the nominal covariance computed by the fixed-threshold filter.

In the conventional data association gating step, the Mahalanobis distance is computed as follows:

(17) $$d_{\mathrm{nom}}^{2}={\tilde{z}}^{⊤}(S^{\mathrm{filter}}{)}^{-1}\tilde{z}.$$

However, the true distribution covariance of the innovation vector $\tilde{z}$ is governed by $S^{\mathrm{true}}$. Because $S^{\mathrm{true}}≻S^{\mathrm{filter}}$, the nominal Mahalanobis distance $d_{\mathrm{nom}}^{2}$ of conventional data association no longer follows a standard $\chi_{2}^{2}$ distribution. The fixed threshold consequently becomes overly stringent, precipitating a sharp escalation in the miss detection rate as Δt increases. To sustain a constant gating detection probability, the threshold must be dynamically compensated.

### 2.4. Time-Interval Adaptive Analysis Oriented to Constellation Revisitation Timeliness

The observation data collected by LEO satellite constellations over moving targets exhibit prominent non-uniform temporal distribution features, where the target detection interval Δt is inextricably linked to the constellation configuration and satellite scale. Due to the intractability of precisely modeling the true kinematic behavior of ships, conventional fixed gating mechanisms introduce a substantial discrepancy between the filter’s predicted states and the actual values. This systematically underestimates the state covariance, ultimately inducing validation matrix sparsification and track fragmentation. To fundamentally rectify the covariance scale mismatch triggered by discontinuous observations, this paper proposes a time-interval adaptive gating method. By establishing an analytical mapping between the satellite detection time interval and the gating threshold, the statistical characteristics of the gating process are strictly conserved across arbitrary observation intervals.

#### 2.4.1. Essential Invariance of Gate Distributions

To quantify the impact of environmental disturbances—such as ocean currents, wind, and unmodeled accelerations—on the innovation covariance, we incorporate the additional mismatch term ΔP(Δt) derived in Section 2.3.2. Here, the parameter $\sigma_{a}^{2}$ explicitly characterizes the intensity of these disturbances, representing the variance of the unmodeled acceleration a (see Section 2.3.1). A larger $\sigma_{a}^{2}$ corresponds to stronger environmental perturbations, which in turn leads to a larger additional covariance contribution $(\sigma_{a}^{2}/4)\Delta t^{4}I_{n_{z}}$. The true innovation covariance is then approximated as:

(18) $$S_{\mathrm{true}}\left(\Delta t\right)=S_{\mathrm{nom}}\left(\Delta t\right)+H\Delta P\left(\Delta t\right)H^{T}\approx S_{\mathrm{nom}}\left(\Delta t\right)+\frac{\sigma_{a}^{2}}{4}\Delta t^{4}I_{n_{z}},$$

where $n_{z}$ denotes the measurement dimension ($n_{z}$ = 2 in this study), and $I_{n_{z}}$ is an $n_{z}\times n_{z}$ identity matrix. The positive semi-definiteness of $S_{\mathrm{true}}(\Delta t)$ is guaranteed by construction: $S_{\mathrm{nom}}(\Delta t)$ is the nominal innovation covariance and is positive semi-definite by definition, while the additional term $(\sigma_{a}^{2}/4)\Delta t^{4}I_{n_{z}}$ is positive definite for any Δt>0 and $\sigma_{a}^{2}>0$. Consequently, $S_{\mathrm{true}}(\Delta t)$ is strictly positive definite, and κ(Δt)≥1 is well-defined.

By introducing a scalar scaling factor κ(Δt)≥1, we assume that the true covariance and the covariance used in conventional association algorithms satisfy a proportional relationship: $S_{\mathrm{true}}(\Delta t)=\kappa (\Delta t)S_{\mathrm{nom}}(\Delta t)$. Whitening the innovation vector as $z=S_{\mathrm{true}}^{-1/2}y_{k}∼N(0,I_{n_{z}})$, the Mahalanobis distance of the conventional association algorithm can be reconstructed as [21]:

(19) $$D_{\mathrm{nom}}^{2}=y_{k}^{T}S_{\mathrm{nom}}^{-1}y_{k}=z^{T}S_{\mathrm{true}}^{1/2}S_{\mathrm{nom}}^{-1}S_{\mathrm{true}}^{1/2}z=\kappa \left(\Delta t\right)\cdot z^{T}z.$$

Since $z^{T}z∼\chi_{n_{z}}^{2}$, it follows that $D_{\mathrm{nom}}^{2}∼\kappa (\Delta t)\cdot \chi_{n_{z}}^{2}$.

The aforementioned analysis demonstrates that TIAG does not alter the essential mathematical characteristics of the chi-squared distribution; rather, it dynamically scales the decision boundary γ(Δt) to synchronize the gating volume with the true uncertainty ellipsoid. Grounded in this property, the conservation of the capture probability for time-interval adaptive gating is formally defined as follows:

**Definition 1.**  *Let the nominal design value of the baseline threshold be* $\gamma_{0}=\chi_{n_{z},P_{G}}^{2}$ *, and let the scaling factor* κ(Δt) *represent the amplification ratio of the true covariance relative to the covariance of the conventional association algorithm at the current sampling epoch. If an adaptive threshold* $\gamma_{\mathrm{adapt}}(\Delta t)=\gamma_{0}\cdot \kappa (\Delta t)$ *is deployed, the probability that a valid measurement passes through the gate is strictly invariant at the pre-specified value* $P_{G}$ *, independent of the observation interval* Δt *:*

(20) $$P\left(D_{\mathrm{nom}}^{2}\leq \gamma_{\mathrm{adapt}}(\Delta t)\right)\equiv P_{G},\forall \Delta t>0.$$

**Proof.**  As derived previously, $D_{\mathrm{nom}}^{2}∼\kappa (\Delta t)\chi_{n_{z}}^{2}$. Substituting the adaptive threshold into the acceptance criterion yields:

(21) $$\begin{array}{cc} P\left(D_{\mathrm{nom}}^{2}\leq \gamma_{0}\kappa \left(\Delta t\right)\right) & =P(\kappa (\Delta t)\chi_{n_{z}}^{2}\leq \gamma_{0}\kappa (\Delta t))\\ & =P(\chi_{n_{z}}^{2}\leq \gamma_{0})\left(∵\kappa \left(\Delta t\right)>0\right).\\ & =F_{\chi_{n_{z}}^{2}}\left(\gamma_{0}\right)\equiv P_{G} \end{array}$$

This completes the proof. □

Definition 1 mathematically guarantees that TIAG satisfies the fundamental prerequisite for JPDA joint event generation; namely, that the probability of the validated measurement set containing the true measurement remains a constant $P_{G}$. At the system level, when the constellation scale expands and the average revisitation cycle shortens, κ(Δt) automatically contracts, preventing an overly loose gate from introducing excessive false alarms. Conversely, when the constellation size shrinks and the average revisitation cycle lengthens,  κ(Δt) automatically expands to maintain continuous track survival.

#### 2.4.2. Derivation of the Adaptive Gating Parameter Expression

Definition 1 establishes the theoretical foundation for the adaptive threshold; consequently, deriving an explicit analytical expression for the scaling factor κ(Δt) enables the engineering implementation of the adaptive gating method. Based on prior derivations, the true innovation covariance augmented by environmental disturbances is approximated as:

(22) $$S_{\mathrm{true}}(\Delta t)\approx S_{\mathrm{nom}}(\Delta t)+\frac{\sigma_{a}^{2}}{4}\Delta t^{4}I_{n_{z}}.$$

Given that $S_{\mathrm{true}}(\Delta t)=\kappa (\Delta t)S_{\mathrm{nom}}(\Delta t)$, by applying a scalar approximation to the matrices, the theoretical expression for the scaling factor is derived as:

(23) $$\kappa \left(\Delta t\right)=1+\frac{\sigma_{a}^{2}\Delta t^{4}}{4S_{\mathrm{nom},\;\mathrm{pos}}\left(\Delta t\right)},$$

where $S_{\mathrm{nom},\;\mathrm{pos}}(\Delta t)$ represents the scalar representative value of the position component in the nominal innovation covariance matrix of the conventional association algorithm, which is taken as its first diagonal element. Under the steady state of the Kalman filter, the position variance term in the predicted covariance $P_{k|k-1}$ is predominantly dictated by the process noise; therefore:

(24) $$S_{\mathrm{nom},\mathrm{pos}}(\Delta t)\approx c_{1}\Delta t^{3}+\sigma_{R}^{2},$$

where $c_{1}$ is a constant related to the process noise intensity q and system parameters, and $\sigma_{R}^{2}$ is the measurement noise variance. When Δt is large, the $\Delta t^{3}$ term dominates, implying $S_{\mathrm{nom},\mathrm{pos}}(\Delta t)\propto \Delta t^{3}$. Substituting this into Equation (24) yields:

(25) $$\kappa \left(\Delta t\right)\approx 1+\frac{\sigma_{a}^{2}}{4c_{1}}\Delta t.$$

In summary, when Δt is large, the theoretical growth order of κ(Δt) is $\Delta t^{1}$, representing a linear growth trend. However, in practical systems, due to the effects of closed-loop feedback and measurement noise, the effective growth slope β typically spans between 1.0∼2.0. To accommodate variations across different constellation topologies and facilitate engineering deployment, a normalized power function is utilized for approximation [25]:

(26) $$\kappa \left(\Delta t\right)={\left(\frac{\Delta t}{T_{\mathrm{ref}}}\right)}^{\beta },$$

where the reference time $T_{\mathrm{ref}}$ is chosen as the minimum constellation revisitation cycle $T_{\mathrm{rev}}$. To prevent false alarm issues under extreme conditions, physical boundaries are introduced into the aforementioned formula:

(1) When the satellite sampling interval satisfies $\Delta t\ll T_{\mathrm{rev}}$, an unconstrained κ→0 would yield an overly narrow gate that induces missed detections. In engineering practice, setting $\Delta t_{min}\approx 0.3T_{\mathrm{rev}}$ yields $\kappa_{min}=0.3^{\beta }\in [0.10,0.20]$;
(2) When the satellite sampling interval satisfies $\Delta t\gg T_{\mathrm{rev}}$, fully extrapolating via $\Delta t^{\beta }$ would encompass a massive amount of clutter. Setting $\Delta t_{max}\approx 2.5T_{\mathrm{rev}}$ yields $\kappa_{max}=2.5^{\beta }\in [3.5,5.0]$. Synthesizing the above derivations, the final expression for the time-interval adaptive gating scaling factor tailored to constellation revisitation timelines is formulated as:
  (27) $$\kappa \left(\Delta t\right)=max\left(\kappa_{min},min\left({\left(\frac{\Delta t}{\Delta t_{\mathrm{ref}}}\right)}^{\beta },\kappa_{max}\right)\right).$$
  Accordingly, the adaptive threshold is generated as $\theta_{\mathrm{base}}=\gamma_{0}\cdot \kappa (\Delta t)$, where $\gamma_{0}$ is the baseline threshold, $\Delta t_{\mathrm{ref}}$ is the reference sampling interval, and β is the attenuation exponent.

#### 2.4.3. Time-Interval Adaptive Gating Threshold Generation Method

The gating expansion factor κ is dynamically computed based on the satellite sampling time interval Δt to generate the baseline validation threshold $\theta_{\mathrm{base}}$ for the current iteration, thereby resolving the uncertainty accumulation problem across prolonged blind zones. The complete procedure is outlined in Table 1 below.

### 2.5. Adaptive Validation Matrix Construction Method

The adaptive validation matrix is designed to quantify the statistical association likelihood between measurements and predicted track states, thereby delineating high-confidence valid association domains. Its core logic involves evaluating the Mahalanobis distance cost matrix C for measurement-track pairs based on the Kalman filter innovation vectors, which is subsequently combined with the adaptive gating mechanism to generate a boolean validation matrix Ω. Therein, the dynamic threshold $\theta_{\mathrm{base}}=\gamma_{0}\cdot \kappa$ dynamically responds to variations in the sampling time interval via a power-law expansion model $\kappa =(\Delta t/\Delta t_{\mathrm{ref}}{)}^{\beta }$, supplemented by a boundary truncation function $max(\kappa_{min},min(\kappa ,\kappa_{max}))$ to prevent threshold overflow.

The innovation of this method lies in breaking through the limitations of conventional fixed thresholds in non-uniform sampling and target maneuvering scenarios. Through a time-interval adaptive threshold evolution strategy, it effectively compensates for the cumulative effect of prediction uncertainty, thereby guaranteeing association completeness.

For the i-th measurement $z_{i}\in R^{m}$ and the predicted state of the *j*-th track ${\hat{x}}_{j|j-1}\in R^{n}$, the innovation vector $\nu_{ij}$ and the innovation covariance matrix $S_{ij}$ are defined as:

(28) $$\nu_{ij}=z_{i}-H{\hat{x}}_{j|j-1},\;\;S_{ij}=HP_{j|j-1}H^{⊤}+R_{i},$$

where H represents the observation matrix, $P_{j|j-1}$ is the predicted error covariance matrix, and $R_{i}$ is the measurement noise covariance. The association cost matrix $C\in R^{M\times N}$ is constructed based on the Mahalanobis distance:

(29) $$C_{ij}=\nu_{ij}^{⊤}S_{ij}^{-1}\nu_{ij}.$$

Considering that fluctuations exist in the actual sensor sampling interval Δt, fixed thresholds are prone to missed associations in dense scenarios or false associations in sparse scenarios. The time-interval adaptive gating scaling factor κ(Δt) is given by Equation (27). Ultimately, the validation matrix $V\in \{0,1{\}}^{M\times N}$ is generated via:

(30) $$V_{ij}=\left\{\begin{array}{cc} 1, & C_{ij}\leq \gamma (\Delta t)\\ 0, & \mathrm{otherwise} \end{array}\right..$$

This matrix fully retains all candidate association pairs that satisfy the statistical significance hypothesis within the current satellite sampling data, providing the foundational topology for subsequent graph-theoretic partitioning. The complete construction procedure for the validation matrix operator is detailed in Table 2.

After the construction of the cost matrix C and the validation matrix Ω, these matrices serve as the foundational input to the subsequent tracking stages. Specifically, the validation matrix Ω is used to build a bipartite graph G that connects measurements and tracks according to their validation status. This graph is then decomposed into connected components via the annealing-based clustering procedure described in Section 3.2, where each connected component forms an independent subproblem for data association. This decomposition effectively limits the combinatorial explosion of JPDA. Meanwhile, the cost matrix C provides the Mahalanobis distances needed for two purposes: (1) it is used by the K-best DFS algorithm in the joint event generation stage (Section 3.3) to rank and select the most probable association hypotheses; and (2) it serves as the weight matrix for the Hungarian hard assignment algorithm, which is invoked as a safety-degradation mechanism when the subproblem size exceeds a pre-specified threshold. Through these mechanisms, the matrices C and Ω directly influence the association quality, computational efficiency, and numerical robustness of the overall tracking system.

## 3. Multi-Target Time-Interval Adaptive Fast Tracking Algorithm for Discontinuous Sparse Observations

### 3.1. Overall Framework

This paper proposes a fast association method for discontinuous sparse observations. Within the JPDA framework, the method incorporates TIAG, annealing-based clustering, heuristic pruning DFS joint event generation, and a hybrid safety-degradation mechanism to balance association accuracy and computational efficiency in sparse detection scenarios.

**Figure 1:**
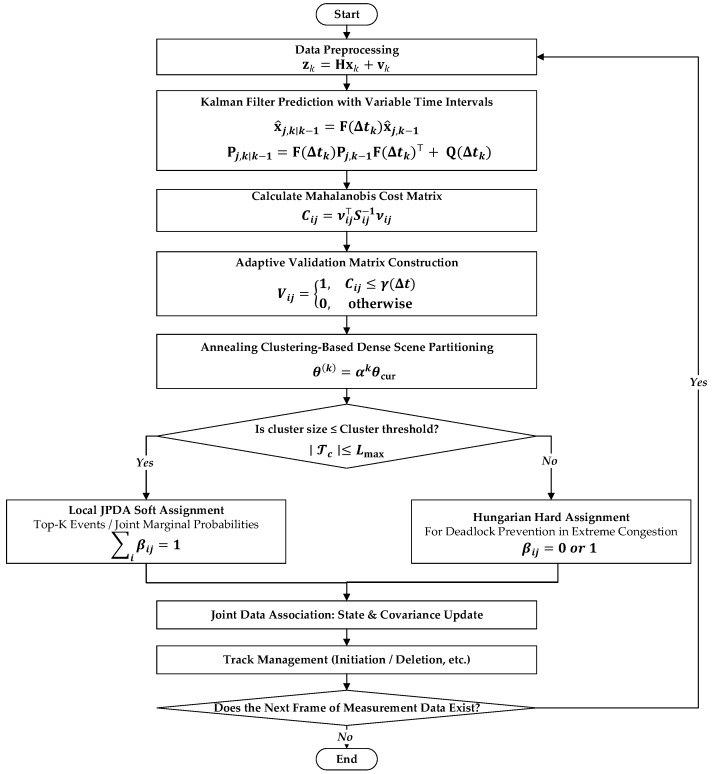
Satellite detection data processing flow.

The complete processing pipeline is illustrated in Figure 1 and described step by step below.

(1) Data Preprocessing: The algorithm starts with the satellite measurement set $D=\{z_{k}^{\left(i\right)}{\}}_{i=1}^{M_{k}}$ received at epoch k. The measurements are preprocessed according to the linear observation model $z_{k}=Hx_{k}+v_{k}$ (Equation (2)), where $v_{k}∼N(0,R)$ represents zero-mean Gaussian measurement noise.
(2) Kalman Filter Prediction with Variable Time Intervals: For each track j, the Kalman filter performs state prediction using the time-varying sampling interval $\Delta t_{k}$. The predicted state and covariance are computed as ${\hat{x}}_{j,k|k-1}=F(\Delta t_{k}){\hat{x}}_{j,k-1}$ and $P_{j,k|k-1}=F(\Delta t_{k})P_{j,k-1}F(\Delta t_{k}{)}^{⊤}+Q(\Delta t_{k})$ (Equations (3) and (4)), where both F(Δt) and Q(Δt) depend on Δt to adapt to non-uniform revisit cycles.
(3) Calculate Mahalanobis Cost Matrix: For each measurement–track pair $\left(i,j\right)$, the innovation vector $\nu_{ij}=z_{i}-H{\hat{x}}_{j|j-1}$ and innovation covariance $S_{ij}=HP_{j|j-1}H^{⊤}+R_{i}$ are first calculated via Equation (28). The Mahalanobis distance $C_{ij}=\nu_{ij}^{⊤}S_{ij}^{-1}\nu_{ij}$ is then computed via Equation (29) for every candidate pair, forming the cost matrix C.
(4) Adaptive Validation Matrix Construction (TIAG Mechanism): To address covariance mismatch caused by discontinuous observations, the TIAG mechanism computes a scaling factor $\kappa (\Delta t)=max(\kappa_{min},min((\Delta t/\Delta t_{\mathrm{ref}}{)}^{\beta },\kappa_{max}))$ via Equation (27), and generates an adaptive threshold $\theta_{\mathrm{base}}=\gamma_{0}\cdot \kappa (\Delta t)$. The boolean validation matrix Ω is then constructed as $V_{ij}=1$ if $C_{ij}\leq \theta_{\mathrm{base}}$, and 0 otherwise (Equation (30)), retaining only those candidate pairs that pass the adaptive gate.
(5) Annealing Clustering-Based Dense Scene Partitioning: In dense scenarios where severe gate overlaps form a giant connected cluster, the annealing-based clustering algorithm recursively partitions the association graph. Starting from $\theta_{\mathrm{cur}}$, the threshold decays via $\theta^{\left(k\right)}=\alpha^{k}\cdot \theta_{\mathrm{cur}}$ until every subcluster satisfies the size constraint $|T_{c}|\leq L_{max}$. The output is a set of independent subclusters $C=\{(D_{1},T_{1}),(D_{2},T_{2}),\ldots ,(D_{K},T_{K})\}$ (Table 3), effectively capping the combinatorial complexity for subsequent JPDA processing.
(6) Hybrid Association Mechanism: For subclusters satisfying $|T_{c}|\leq L_{max}$, the algorithm performs local JPDA soft assignment, computing marginal association probabilities $\beta_{ij}$ which satisfy $\sum_{i}\beta_{ij}=1$ for each target. A K-best DFS heap pruning strategy retains only the top K=1000 joint events. If a subcluster still exceeds $L_{max}$, the Hungarian algorithm is triggered as a safety fallback, yielding binary association probabilities $\beta_{ij}\in \{0,1\}$.
(7) Joint Data Association: Once the association weights $\beta_{ij}$ are obtained, the combined innovation is computed as ${\tilde{z}}_{j}=\sum_{i}\beta_{ij}\cdot {\tilde{z}}_{ij}$. The Kalman gain is calculated as $K_{j}=P_{j,k|k-1}H^{⊤}S_{j}^{-1}$, and the state and covariance are updated to ${\hat{x}}_{j,k|k}$ and $P_{j,k|k}$ via the standard JPDA update equations.
(8) Track Management and Iteration: Finally, the track management module performs track initiation, confirmation, and deletion based on association histories and track quality metrics. If the next frame of measurement data exists, the algorithm returns to Step (2) and iterates; otherwise, the tracking loop terminates.

**Table 3:** The Annealing-Based Clustering Method for Dense Scenarios.

Detailed Steps of the Algorithm
**Input:** Track set $T=\{t_{1},t_{2},\ldots ,t_{N}\}$ and measurement set $D=\{d_{1},d_{2},\ldots ,d_{M}\}$; Baseline gating coefficient $\gamma_{0}$ and time-interval adaptive expansion function κ(Δt); Annealing decay factor α∈(0,1) and lower-bound threshold coefficient $\alpha_{min}\in (0,1)$; Maximum track capacity constraint per subcluster $L_{max}$ ($L_{max}=8$ in the engineering implementation of this study). **Output:** Independent subcluster set satisfying the scale constraints $C=\{(D_{1},T_{1}),(D_{2},T_{2}),\ldots ,(D_{K},T_{K})\}$ **Steps:** **// ================= Initialization Phase ===================** : $\theta_{\mathrm{cur}}\leftarrow \gamma_{0}\cdot \kappa (\Delta t)$ : $\theta_{min}\leftarrow \alpha_{min}\cdot \theta_{\mathrm{cur}}$ : $\Omega \leftarrow \mathrm{BuildValidationMatrix}(T,D,\theta_{\mathrm{cur}})$ : G←BipartiteGraph(T∪D,Ω) : C←ConnectedComponents(G) **// ================ Iterative Annealing Splitting ===============** 6. : **while** $\exists (D_{c},T_{c})\in C$ satisfying $|T_{c}|>L_{max}$ and $\theta_{\mathrm{cur}}>\theta_{min}$ **do** 7. : $\theta_{\mathrm{cur}}\leftarrow \alpha \cdot \theta_{\mathrm{cur}}$ 8. : $C_{\mathrm{new}}\leftarrow \emptyset$ 9. : **for each** $(D_{c},T_{c})\in C$ **do** 10. : **if** $|T_{c}|\leq L_{max}$ **then** 11. : $C_{\mathrm{new}}\leftarrow C_{\mathrm{new}}\cup \{(D_{c},T_{c})\}▹$ *Scale target reached* 12. **else** 13. $\;\Omega_{\mathrm{sub}}\leftarrow \mathrm{BuildValidationMatrix}(T_{c},D_{c},\theta_{\mathrm{cur}})$ 14. $\;G_{\mathrm{sub}}\leftarrow \mathrm{BipartiteGraph}(T_{c}\cup D_{c},\Omega_{\mathrm{sub}})$ 15. $\;\{(D_{c}^{\left(k\right)},T_{c}^{\left(k\right)})\}\leftarrow \mathrm{ConnectedComponents}(G_{\mathrm{sub}})$ 16. $\;C_{\mathrm{new}}\leftarrow C_{\mathrm{new}}\cup \{(D_{c}^{\left(k\right)},T_{c}^{\left(k\right)})\}$ 17. **end if** 18. **end for** 19. $\;C\leftarrow C_{\mathrm{new}}$ **20.** **end** **while**

The primary innovations are summarized as follows:

(1) Time-interval Adaptive Gating Mechanism: An analytical mapping between the satellite observation time intervals and the gating thresholds is derived, and an adaptive threshold tuning strategy based on a temporal scaling factor is proposed, thereby effectively resolving the validation gate failure problem induced by discontinuous observations.
(2) Annealing-Based Clustering Algorithm: To resolve the massive connected cluster problem, a progressive graph partitioning algorithm predicated on an annealing strategy is presented. By incrementally decaying the validation thresholds, a highly uncertain, dense association graph is recursively decomposed into several manageable subgraphs, which simultaneously guarantees association completeness and caps computational complexity.
(3) Hybrid Degradation Safety Mechanism: A hybrid strategy combines K-best DFS heap pruning with the Hungarian algorithm, switching to hard-assignment mode when cluster size exceeds a threshold to ensure numerical stability in extreme cases.

### 3.2. Clustering Design for Dense Scenarios Based on Annealing Clustering

Under sparse observation conditions, the computational complexity of the Joint Probabilistic Data Association method scales exponentially with the number of targets and measurements, presenting a primary bottleneck for real-time multi-target tracking. In conventional sparse target detection scenarios, connected component decomposition can achieve effective partitioning. However, when the spatial distribution of targets is dense and the observation intervals are large, the validation gates overlap severely, causing the validation matrix to become dense and consequently forming a “giant cluster” that encompasses almost all targets and measurements, which renders the partitioning strategy completely ineffective [26]. To address this issue, this section proposes an adaptive partitioning association algorithm predicated on an annealing strategy. By incrementally decaying the gating thresholds, the giant cluster is recursively decomposed into several subclusters of manageable sizes, thereby effectively capping the computational complexity while preserving the completeness of the association information.

In the classical Multi-Target Tracking (MTT) framework, Joint Probabilistic Data Association (JPDA) has emerged as one of the baseline algorithms for dense clutter environments due to its capability to explicitly handle the ambiguities in measurement-to-track assignments. However, the underlying computation of JPDA is fundamentally a combinatorial optimization problem. Given M valid measurements and N confirmed tracks, the cardinality of the joint association event space equals the number of all legal mappings that assign all measurements to N+1 sources (N true targets plus 1 clutter source). This combinatorial number explodes super-exponentially with the growth of M and N, and its asymptotic complexity can be expressed as $O((N+1{)}^{M})$. When both M and N scale to a moderate size (e.g., N,M≥15), the cardinality of the hypothesis space exceeds the order of $10^{9}$, rendering exact enumeration and probability normalization completely computationally intractable in real-time systems.

To resolve the giant cluster problem, and inspired by the philosophy of “high-temperature exploration and low-temperature convergence” inherent in the simulated annealing algorithm, this paper proposes a Time-Interval Annealing-based Clustering algorithm. Its core concept is based on the time-interval adaptive gating threshold; by incrementally decaying the gating threshold, the giant cluster is progressively segmented into several subclusters of manageable sizes, thereby achieving controllable computational complexity while preserving the completeness of the association information.

In extremely dense scenarios, an initial wide gate threshold may cause the entire scenario to collapse into a single giant connected component. To address this, the algorithm introduces the “cooling” mechanism from the concept of Simulated Annealing. An annealing threshold sequence is defined as $\theta^{\left(k\right)}=\alpha^{k}\theta_{\mathrm{cur}}$, where α∈(0,1) denotes the decay factor. The annealing process is initialized with the adaptive gating threshold obtained from Section 2. Specifically, the initial threshold $\theta_{\mathrm{cur}}$ in Table 3 is set as $\theta_{\mathrm{cur}}=\gamma_{0}\cdot \kappa (\Delta t)$, where κ(Δt) is the time-interval adaptive scaling factor derived in Equation (27). This ensures that the clustering algorithm operates on a validation matrix Ω constructed from the adaptively scaled gate, establishing a direct connection between the theoretical analysis of covariance mismatch (Section 2.3) and the practical clustering decomposition (Section 3.2). As the iterations progress, the threshold gradually tightens, and the originally connected graph structure undergoes topological splitting due to the elimination of weakly associated edges. Consequently, the giant cluster is physically partitioned into multiple micro-subclusters that satisfy the computational capacity constraint ($|T_{c}|\leq L_{max}$).

The core concept of the annealing-based clustering algorithm is to recursively segment the giant connected cluster—originally formed by gating overlaps—into several subclusters of manageable sizes by gradually reducing the gating threshold. In each iteration, the algorithm first re-evaluates the association possibilities between all tracks and measurements based on the current gating threshold to construct a boolean validation matrix via the BuildValidationMatrix function. Subsequently, this matrix is treated as the adjacency relationship of a bipartite graph using the BipartiteGraph function. Through the connected component decomposition algorithm in graph theory, independent subgraphs are identified using the ConnectedComponents function, where each subgraph corresponds to a subproblem that can undergo data association independently. These two steps are executed alternately until the sizes of all subclusters satisfy the pre-specified upper bound or the threshold drops to its lower bound. Below, the specific implementations of the validation matrix construction method and the connected component decomposition are elaborated respectively.

### 3.3. Hybrid Safety-Degradation Mechanism

The joint event generation procedure in this section relies on the cost matrix C and validation matrix Ω constructed in Section 2.5. The cost matrix C, which stores the Mahalanobis distances between measurements and tracks as defined in Equation (29), serves as the input to the K-best DFS algorithm for ranking and selecting high-probability association hypotheses. Additionally, the same matrix C is used by the Hungarian algorithm when the safety-degradation mechanism is triggered, providing the weights for the minimum-weight matching that yields a deterministic one-to-one association. In this way, the adaptive gating and validation matrix construction from Section 2 directly support the joint event generation and degradation mechanisms in Section 3.

Based on the local subclusters partitioned via annealing-based clustering, this paper further incorporates a heuristic pruning strategy to construct an efficient joint event search mechanism that balances both tracking accuracy and computational efficiency. Even when subjected to spatial clustering constraints that strictly cap individual subcluster scales within n≤8  and m≤8, the total number of legal joint events under exhaustive enumeration can still scale up to the order of $\left(n+1{)}^{m}\approx 9^{8}\right.$. Such a massive computational burden severely undermines the real-time performance of the tracking system, making it difficult to satisfy the engineering deployment requirements of high-speed and high-frequency scenarios [27]. To address this bottleneck, this paper adopts a *K*-best joint event approximation strategy [28]. The theoretical premise of this strategy is that in the vast majority of practical tracking scenarios, only a tiny fraction of high-probability association hypotheses dominate the posterior state estimation, whereas the weighted contribution of the remaining sea of low-probability hypotheses to the filtering results is negligible. Consequently, the algorithm retains only the *K* = 1000 events with the highest global probabilities for subsequent processing, achieving an order-of-magnitude compression in computational complexity while keeping the loss of accuracy well within a controllable range.

Nevertheless, despite the annealing-based clustering’s efforts to restrict cluster sizes within $L_{max}\leq 8$, the annealing algorithm may fail to achieve sufficient segmentation under extreme scenarios, such as when all targets and measurements completely overlap or during the first update following a prolonged period without observations. Under such conditions, if the system continues to employ the K-best JPDA, it may still suffer from deadlocks induced by combinatorial explosion. Therefore, a Hungarian algorithm-based hard assignment is introduced as a safe fallback mechanism [29,30]. The Hungarian algorithm, also known as the Kuhn–Munkres algorithm, is a combinatorial optimization method that solves the assignment problem in polynomial time [30]—given a cost matrix of measurement–track pairs, it finds the one-to-one assignment that minimizes the total cost. In this work, when the annealing-based clustering fails to reduce a subproblem below the size limit, the Hungarian algorithm is invoked to produce a deterministic hard assignment using the Mahalanobis distance cost matrix C from Section 2.5. When the number of tracks in a specific subcluster exceeds the maximum cluster threshold, the soft probabilistic association is bypassed. Instead, the algorithm directly deploys the Hungarian algorithm to solve for the minimum-weight matching of the cost matrix (Mahalanobis distance), yielding a deterministic one-to-one association and setting the corresponding joint association probability $\beta_{i,j}$ to 1. This degradation strategy guarantees that the algorithm returns results within a bounded duration under any circumstances, thereby ensuring system-level robustness.

## 4. Simulation Design and Result Analysis

### 4.1. Simulation Design

To comprehensively validate the tracking performance of the adaptive-threshold annealing-clustering JPDA algorithm in multi-target, dense clutter environments, this study constructs a space-based multi-sensor non-uniform detection and tracking simulation scenario utilizing STK (Systems Tool Kit). The scenario matrix is designed to cover three typical low-Earth-orbit (LEO) constellation configurations of varying scales alongside two density tiers of maritime moving targets. This setup serves to systematically investigate the adaptability of the proposed algorithm across diverse satellite coverage characteristics and target densities.

#### 4.1.1. Task Region and Target Motion Model

The simulation region is designated within the Western Pacific Ocean (34° N~39° N, 146° E~152° E), covering international core maritime traffic lanes characterized by typical multi-target intersections and dense shipping routes. The target fleet is configured across two tiers of ship density: low density (15 ships) and high density (30 ships). The motion model for these ocean-going ships is defined as constant velocity (CV) linear motion, spanning a total trajectory generation timeline of 24 h. The kinematic trajectory of each ship is governed by its initial position, initial velocity, heading angle, and minor random accelerations. The kinematic parameters of the moving targets are summarized in Table 4. The generated ground-truth trajectories are stored in JSON format, capturing the true latitude, longitude, and velocity at each discrete time epoch. 

#### 4.1.2. Constellation Parameters and Simulation Procedure

This study adopts the Walker-Delta constellation configuration to design three representative satellite deployment schemes. These configurations differ in orbital altitude, satellite count, and revisitation characteristics. A stable spatial topology is generated for each of the three configurations using the STK orbit simulation module to achieve continuous coverage and alternating observations over the target maritime domain. Utilizing the STK Access module, the spatial visibility and detection time windows of the satellites relative to the maritime moving targets are precisely computed. Once a satellite completes a detection task, the observation data is downlinked in real time to the ground station, where centralized data association fitting is executed via track association algorithms.

**Figure 2:**
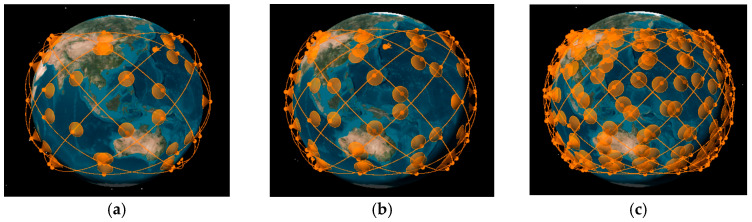
Three types of Walker-Delta constellation configurations: (**a**) Configuration A; (**b**) Configuration B; (**c**) Configuration C.

During the satellite observation process, the measurement errors of the satellite payloads strictly follow a Gaussian distribution, and the standard deviation of the position noise is uniformly configured across the different constellation configurations. To approximate a realistic and complex maritime detection environment, uniformly distributed random clutter points are injected across the entire monitored region based on areal density. This setup simulates multiple interference sources, including sea clutter, false alarms, and electromagnetic interference. The clutter intensity increases synchronously with the frequency at which the targets are detected, and the number of clutter points generated during each satellite observation is the product of the clutter density and the area of the observed maritime region. The specific design parameters of the constellations are detailed in Table 5. The three constellation configurations are illustrated in Figure 2.

### 4.2. Design of the Evaluation Metrics System

#### 4.2.1. Track Match Success Rate

The Track Match Success Rate (SR) is a core metric utilized to evaluate the track maintenance capability and association robustness of multi-target tracking algorithms within complex scenarios. Distinct from the Root Mean Square Error (RMSE), which exclusively focuses on instantaneous position accuracy, the SR metric quantifies—from a trajectory-level perspective—whether an algorithm can continuously and correctly bind fused tracks to true targets amidst dense target crossings. Consequently, it measures the algorithm’s capacity to avoid typical failure modes such as track swaps, false tracks, and track loss.

(1) Single-Track Match Criterion

For a specific fused track $T_{f}$ and a true trajectory $T_{t}$, their average spherical distance is defined as:

(31) $$\overset{-}{d}\left(T_{f},T_{t}\right)=\frac{1}{M}\sum_{j=1}^{M}d_{H}\left(p_{j}^{f},p_{j}^{t}\right),$$

where M denotes the number of valid matching frames between the two trajectories along the time axis (i.e., frames where both the measurement and the ground truth coexist); $p_{j}^{f}=[{\mathrm{lat}}_{j}^{f},{\mathrm{lon}}_{j}^{f}{]}^{⊤}$ and $p_{j}^{t}=[{\mathrm{lat}}_{j}^{t},{\mathrm{lon}}_{j}^{t}{]}^{⊤}$ represent the fused position and the true position at the *j*-th frame, respectively; $d_{H}\left(\cdot \right)$ denotes the Haversine spherical distance formula, which precisely calculates the great-circle distance under the influence of Earth’s curvature. Given a pre-specified spatial threshold δ, the binary match function is defined as:

(32) $$M\left(T_{f},T_{t};\delta \right)=\left\{\begin{array}{cc} 1 & if\overset{-}{\;d}\left(T_{f},T_{t}\right)\leq \delta \;and\;M\geq M_{min}\\ 0 & otherwise \end{array}\right..$$

In the simulation, the minimum required valid frames is configured as $M_{min}=3$ to filter out pseudo-matches resulting from brief, coincidental overlaps.

(2) Global Match Success Rate Calculation

Let the true target set be $T=\{T_{1}^{t},T_{2}^{t},\ldots ,T_{N}^{t}\}$ and the fused track set be $F=\{T_{1}^{f},T_{2}^{f},\ldots ,T_{K}^{f}\}$. By constructing a bipartite graph matching matrix $A\in \{0,1{\}}^{K\times N}$, where $A_{ij}=M(T_{i}^{f},T_{j}^{t};\delta )$, the Hungarian algorithm is deployed to solve for the maximum cardinality matching. This ensures that each true target is matched to at most one fused track, thereby satisfying the one-to-one constraint [31,32]. The final track match success rate is defined as:

(33) $$SR=\frac{N_{matched}}{N_{true}}\times 100\%=\frac{\sum_{j=1}^{N}{max}_{i}A_{ij}}{N}\times 100\%.$$

This metric directly reflects the tracking completeness of the algorithm at the target level. SR = 100% indicates that all true targets are correctly tracked, whereas SR < 100% implies the occurrence of target loss or track confusion. In typical scenarios involving target formation sailing or cross-overs, a high match success rate signifies that the algorithm can effectively suppress track swaps and the generation of false tracks.

#### 4.2.2. Root Mean Square Error

The Root Mean Square Error (RMSE) is a core metric utilized to evaluate the accuracy of tracking algorithms [21,33], serving to quantify the spatial deviation between fused tracks and true trajectories. Its mathematical definition is expressed as:

(34) $$\mathrm{RMSE}=\sqrt{\frac{1}{N}\sum_{i=1}^{N}d_{H}^{2}\left(p_{i}^{fused},p_{i}^{true}\right)},$$

where N denotes the total number of effectively matched measurement-track point pairs; $p_{i}^{fused}=[{\mathrm{lat}}_{i}^{f},{\mathrm{lon}}_{i}^{f}{]}^{T}$ and $p_{i}^{true}=[{\mathrm{lat}}_{i}^{t},{\mathrm{lon}}_{i}^{t}{]}^{T}$ represent the geographic latitude and longitude coordinates of the fused track point and the corresponding true trajectory point, respectively; $d_{H}(\cdot )$ denotes the Haversine spherical distance formula, which precisely calculates the great-circle distance between two points under the influence of Earth’s curvature:

(35) $$d_{H}=2R\cdot arcsin\left(\sqrt{{sin}^{2}\left(\frac{\Delta \phi }{2}\right)+cos\phi_{1}cos\phi_{2}{sin}^{2}\left(\frac{\Delta \lambda }{2}\right)}\right).$$

In this formula, R=6371 km represents the average radius of the Earth, ϕ denotes the latitude, and λ denotes the longitude.

The RMSE directly reflects the capability of the filtering and association mechanisms to suppress raw measurement noise. In the simulation, the RMSE of the raw measurement errors is adopted as the baseline. By comparing the RMSE reduction percentage achieved after fusion across different algorithms, the accuracy gain brought by data association can be quantitatively evaluated. A smaller RMSE value indicates higher tracking accuracy. To eliminate the impact of random noise, each set of experiments is executed 30 times repeatedly, and the average value is taken [34].

#### 4.2.3. Computational Execution Time

This metric records the wall-clock time consumed by the data association and tracking algorithm from loading the preprocessed measurement data (obtained offline from STK simulations) to the complete output of the fused tracks, quantified in seconds. The STK simulation itself is performed offline prior to the tracking stage and is excluded from this timing measurement, as the focus is on the real-time performance of the proposed data association method rather than the scenario generation process.

To guarantee the objectivity of the test results, all experiments are executed under a unified hardware and software environment: 12th Gen Intel(R) Core (TM) i7-1260P (2.10 GHz), 16 GB RAM, Windows 11 operating system, using Python (v3.9) for single-threaded computing. For each experimental configuration, the simulation is run 30 times independently and repeatedly, and the median is taken as the final execution time data.

Computational execution time is the core benchmark for evaluating the real-time performance of an algorithm in practical engineering deployment. In constrained deployment scenarios such as on-board satellite platforms or edge computing nodes, the algorithm must complete data processing within a strict time window; otherwise, it will induce track update delays, thereby undermining the decision-making efficiency of closed-loop command and control systems [35,36]. In this study, the timeout threshold is configured as $T_{max}=600\;s$. Once this limit is exceeded, the execution is forcibly terminated and flagged as a “timeout failure”.

### 4.3. Simulation Results and Analysis

#### 4.3.1. Effectiveness Analysis of the Time-Interval Adaptive Gating Mechanism

To evaluate the TIAG mechanism under varying constellation coverage, this section presents comparative experiments with 15 moving targets. The evaluations are performed across three distinct deployment scales: a sparse constellation (Configuration A: 8-8-2, 64 satellites), a baseline constellation (Configuration B: 10-10-2, 100 satellites), and a dense constellation (Configuration C: 15-15-2, 225 satellites). The performance discrepancies among four tracking algorithms—the Hungarian hard assignment algorithm with a fixed gating threshold (HUN-Fix), the Hungarian hard assignment algorithm with a time-interval adaptive gating threshold (HUN-Adp), the joint probabilistic data association algorithm based on simulated annealing clustering with a fixed gating threshold (JPDA-AC-Fix), and the proposed joint probabilistic data association algorithm based on simulated annealing clustering with a time-interval adaptive gating threshold (JPDA-AC-Adp)—are comprehensively analyzed across three dimensions: statistical Root Mean Square Error (RMSE) position accuracy, frame-by-frame error evolution, and track match success rate. These metrics evaluate TIAG in terms of association region modulation, clutter suppression, track continuity, and tracking accuracy.

(1) Algorithm Performance Comparison in Sparse Constellation Scenarios

In Configuration A (the 8/8/2 sparse constellation), the measurement data exhibits pronounced non-uniformity due to sparse satellite coverage and prolonged revisit intervals, which inherently causes conventional fixed-threshold strategies to suffer from missing valid measurements and tracking association failures.

**Figure 3:**
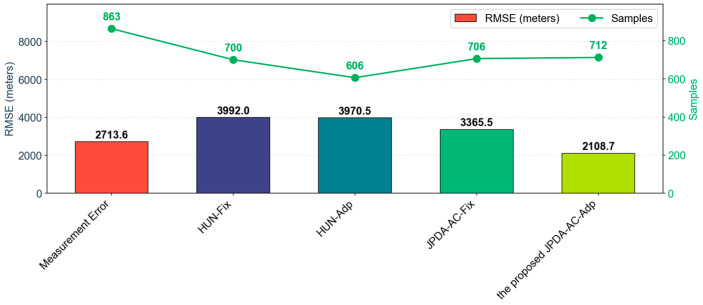
Comparison of position root mean square errors (RMSE) and sample sizes among different algorithms under the 8/8/2 sparse constellation scenario.

The statistical RMSE results (Figure 3) indicate that the baseline measurement error is 2713.6 m. The fixed-threshold Hungarian algorithm (HUN-Fix) yields an RMSE of 3992.0 m, which significantly exceeds the raw observation baseline and indicates a severe performance degradation in data association. The adaptive-threshold Hungarian algorithm (HUN-Adp) slightly improves the RMSE to 3976.5 m, while maintaining a relatively stable number of effective samples (706), yet its tracking accuracy remains poor. In contrast, the fixed-threshold simulated annealing-clustering JPDA algorithm (JPDA-AC-Fix) achieves an RMSE of 3365.5 m, demonstrating the benefit of probabilistic data association over hard assignment. More importantly, its adaptive-threshold counterpart, the JPDA-AC-Adp algorithm, further drives the RMSE down to 2108.7 m, while simultaneously maintaining a high number of effective measurement samples (712), thereby achieving a substantial leap forward in tracking accuracy and system robustness.

**Figure 4:**
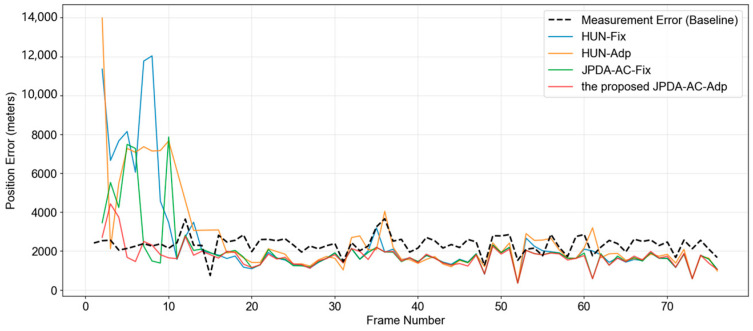
Frame-by-frame average tracking position error curves among different algorithms under the 8/8/2 sparse constellation scenario.

The frame-by-frame average tracking error curves (Figure 4) reveal that fixed-threshold algorithms are highly susceptible to massive error spikes during specific satellite sampling epochs characterized by target cross-overs or localized clutter surges. Conversely, the adaptive-threshold mechanism dynamically scales the validation regions to effectively dampen these anomalous fluctuations, ensuring a much smoother and more stable error profile throughout the entire trajectory timeline.

**Figure 5:**
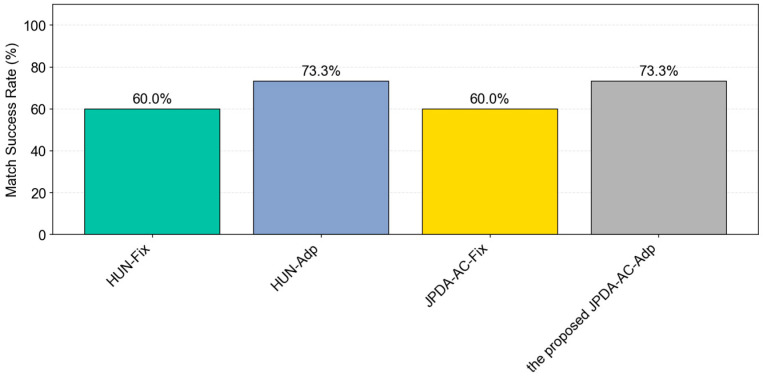
Comparison of track match success rates among different algorithms under the 8/8/2 sparse constellation scenario.

The trajectory-level track match success rates (Figure 5) further substantiate these findings. The track match success rate for HUN-Fix stands at 60.0%, while HUN-Adp achieves 73.3%. The JPDA-AC-Fix algorithm attains 60.0%, whereas the proposed JPDA-AC-Adp algorithm achieves a substantially higher track maintenance rate of 73.3%, demonstrating its superior association robustness and track continuity in sparse observation scenarios.

For sparse constellation detection, TIAG improves the tracking accuracy, error stability, and track maintenance of the annealing-clustering JPDA approach. The JPDA-AC-Fix algorithm already outperforms the Hungarian-based approaches, and the adaptive-threshold JPDA-AC-Adp algorithm delivers the optimal comprehensive performance, establishing it as the most effective system configuration for multi-target tracking under sparse satellite coverage.

(2) Algorithm Performance Comparison in Baseline Constellation Scenarios

In Configuration B (the 10/10/2 baseline constellation), where the satellite coverage is uniform and the revisit intervals are moderate, the time-interval adaptive gating strategy achieves robust optimization of the tracking performance.

**Figure 6:**
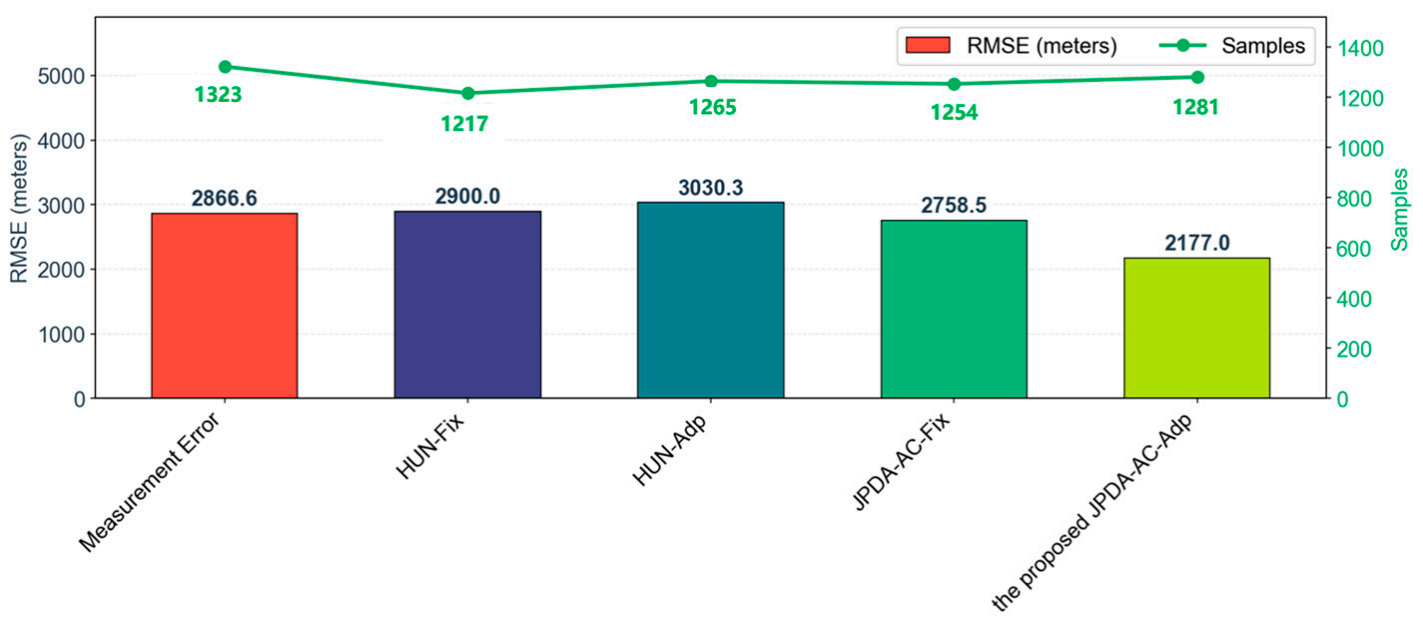
Comparison of position root mean square errors (RMSE) among different algorithms under the 10/10/2 baseline constellation scenario.

The statistical RMSE results (Figure 6) indicate that the baseline measurement error is 2866.6 m. The fixed-threshold Hungarian hard assignment algorithm (HUN-Fix) yields an RMSE of 2900.0 m, which is comparable to the observation baseline, indicating limited improvement from data association. The adaptive-threshold Hungarian algorithm (HUN-Adp) produces an RMSE of 3030.3 m, slightly worse than the raw measurement error, with 1265 effective samples, suggesting that the adaptive gating mechanism offers only marginal benefit for hard-assignment methods in this scenario. In contrast, the fixed-threshold simulated annealing-clustering JPDA algorithm (JPDA-AC-Fix) achieves an RMSE of 2758.5 m, outperforming the Hungarian-based approaches. More importantly, its adaptive-threshold counterpart, the JPDA-AC-Adp algorithm, significantly reduces the RMSE to 2177.0 m while maintaining a high number of effective measurement samples (1281), achieving a substantial improvement in tracking accuracy.

**Figure 7:**
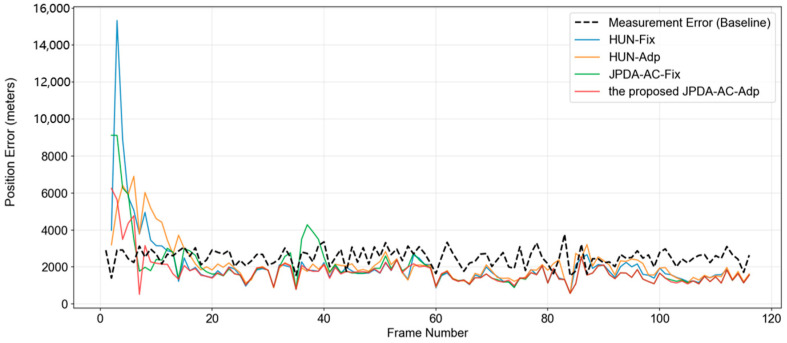
Frame-by-frame average tracking position error curves among different algorithms under the 10/10/2 baseline constellation scenario.

The frame-by-frame tracking error curves (Figure 7) reveal that Hungarian-based approaches (particularly HUN-Adp) suffer from large error spikes during target initialization and cross-over phases, whereas the adaptive gating mechanism effectively mitigates these anomalous fluctuations. Under the simulated annealing-clustering JPDA framework, incorporating the adaptive gating mechanism (JPDA-AC-Adp) guarantees a much smoother and lower error profile throughout the entire trajectory timeline, demonstrating superior dynamic stability and clutter suppression capability.

**Figure 8:**
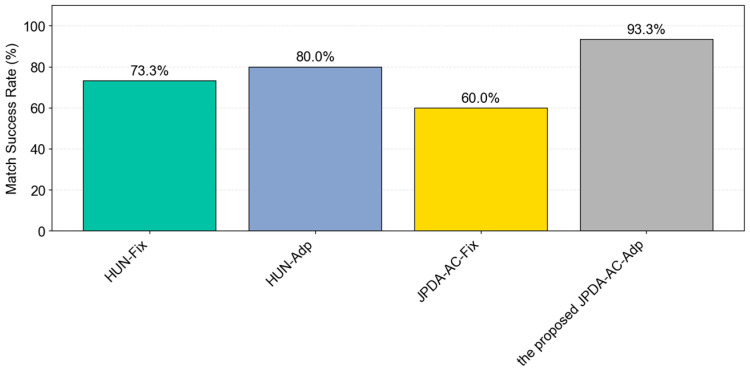
Comparison of track match success rates among different algorithms under the 10/10/2 baseline constellation scenario.

In terms of trajectory-level track match success rates (Figure 8), HUN-Fix achieves 73.3%, and HUN-Adp achieves 80.0%, indicating that the Hungarian methods maintain moderate track continuity in the baseline constellation scenario. JPDA-AC-Fix attains only 60.0%, lower than the Hungarian methods, which may suggest that the fixed-gating parameters combined with annealing clustering lead to some track deletions during certain periods under baseline constellation conditions. In contrast, the proposed JPDA-AC-Adp algorithm achieves a significantly higher success rate of 93.3%, verifying the critical role of the adaptive gating mechanism in enhancing track continuity.

In summary, for baseline constellation detection scenarios, the time-interval adaptive gating mechanism effectively suppresses tracking divergence, reduces position errors, and enhances tracking smoothness. Although JPDA-AC-Fix under the fixed threshold underperforms the Hungarian methods in terms of success rate, the adaptive-threshold JPDA-AC-Adp algorithm delivers the optimal comprehensive performance among all benchmarked configurations, with superior accuracy, stability, and track completeness, making it highly suitable for multi-target tracking in dense clutter environments.

(3) Algorithm Performance Comparison in Dense Constellation Scenarios

In Configuration C (the 15/15/2 dense constellation), the satellites feature rapid revisitation and a high degree of measurement redundancy. Under these conditions, conventional fixed-threshold strategies are highly susceptible to introducing redundant measurements that induce association confusion. Conversely, the time-interval adaptive gating strategy enables precise pruning of clutter and invalid hypotheses.

**Figure 9:**
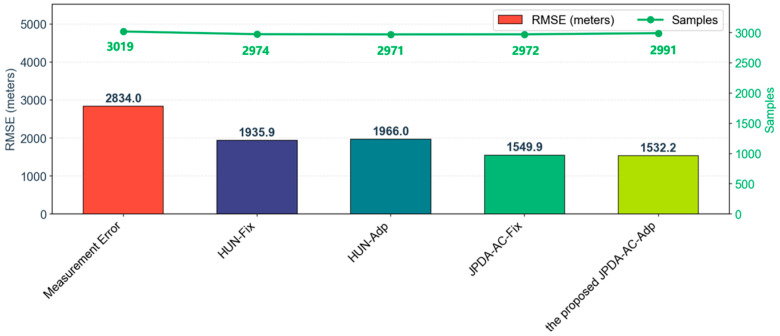
Comparison of position root mean square errors (RMSE) and sample sizes among different algorithms under the 15/15/2 dense constellation scenario.

The statistical RMSE results (Figure 9) indicate that the baseline measurement error is 2834.0 m. The fixed-threshold Hungarian hard assignment algorithm (HUN-Fix) yields an RMSE of 1935.9 m, significantly outperforming the raw observation baseline and demonstrating the benefit of dense coverage. Its adaptive-threshold counterpart (HUN-Adp) achieves a comparable RMSE of 1966.0 m, with a similar number of effective samples (2971), indicating that adaptive gating offers marginal additional benefit for Hungarian hard assignment in this dense scenario. In contrast, the joint probabilistic data association algorithm based on simulated annealing clustering with a fixed gating threshold (JPDA-AC-Fix) achieves a substantially lower RMSE of 1549.9 m. The proposed adaptive-threshold version (JPDA-AC-Adp) further improves the tracking accuracy to 1532.2 m, while simultaneously maintaining a higher effective sample count (2991), thereby achieving the best tracking accuracy among all benchmarked algorithms.

**Figure 10:**
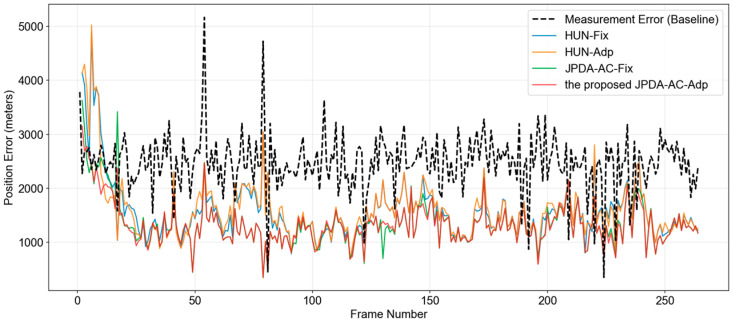
Frame-by-frame average tracking position error curves among different algorithms under the 15/15/2 dense constellation scenario.

The frame-by-frame tracking error curves (Figure 10) reveal that fixed-threshold algorithms still suffer from noticeable error fluctuations during dense target cross-over phases. In contrast, the adaptive gating mechanism dynamically constricts the validation regions to effectively suppress redundant measurements and localized clutter interference. Consequently, its entire error profile remains significantly smoother across the trajectory timeline, dramatically minimizing anomalous extreme error spikes.

**Figure 11:**
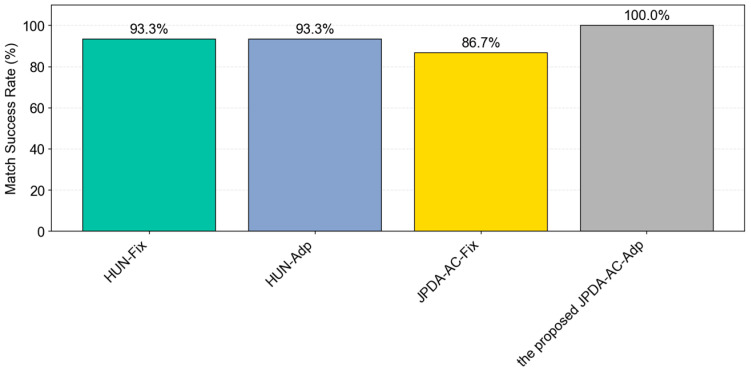
Comparison of track match success rates among different algorithms under the 15/15/2 dense constellation scenario.

The trajectory-level track match success rates (Figure 11) further substantiate these findings. HUN-Fix and HUN-Adp both achieve a high success rate of 93.3%, demonstrating that Hungarian hard assignment performs well in dense coverage scenarios. JPDA-AC-Fix attains 86.7%, which is slightly lower than the Hungarian-based methods under fixed-gating conditions. In sharp contrast, the proposed adaptive-threshold JPDA-AC-Adp configuration successfully achieves a perfect track maintenance rate of 100.0%, highlighting its superior association robustness and the critical role of adaptive gating in ensuring complete track continuity even in dense clutter environments.

In summary, for dense constellation detection scenarios, the time-interval adaptive gating mechanism significantly enhances the tracking accuracy, error smoothness, and track maintenance capability of the annealing-clustering JPDA approach. While JPDA-AC-Fix under a fixed threshold already outperforms the Hungarian-based methods in terms of RMSE, its success rate (86.7%) falls slightly behind HUN-Adp (93.3%), suggesting that the performance of the annealing clustering approach is more sensitive to the fixed-threshold setting in dense scenarios. Once combined with the adaptive gating mechanism, JPDA-AC-Adp delivers the optimal comprehensive performance among all benchmarked configurations, achieving the lowest RMSE (1532.2 m) and the highest track match success rate (100.0%). It therefore completely satisfies the robust and stable all-target tracking requirements in high-coverage, high-density clutter environments.

#### 4.3.2. Comparative Analysis of Algorithmic Association Effectiveness in Dense Scenarios

**Figure 12:**
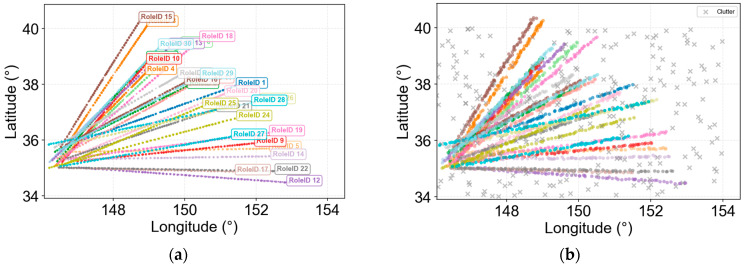
Satellite detection views in the high-density tracking scenario: (**a**) Ground truth; (**b**) Measurement tracks.

Utilizing the Walker-Delta configuration B (10/10/2) baseline constellation as the evaluation platform, this section evaluates the association effectiveness of the tracking algorithms within a typical dense scenario featuring 30 moving targets. The satellite detection views for this high-density scenario are illustrated in Figure 12. A comprehensive comparative analysis is conducted between the Hungarian hard assignment algorithm with a time-interval adaptive gating threshold (HUN-Adp) and the proposed joint probabilistic data association algorithm based on simulated annealing clustering with a time-interval adaptive gating threshold (JPDA-AC-Adp) across four distinct dimensions: statistical position error, temporal error evolution, track match success rate, and computational execution time. Furthermore, the joint probabilistic data association algorithm with time-interval adaptive gating and K-best pruning (JPDA-K-Adp) is incorporated into the computational efficiency evaluation. This comprehensive evaluation serves to unveil the critical discrepancies in tracking performance and engineering applicability among different algorithms under conditions of high target density and heavy clutter interference.

**Figure 13:**
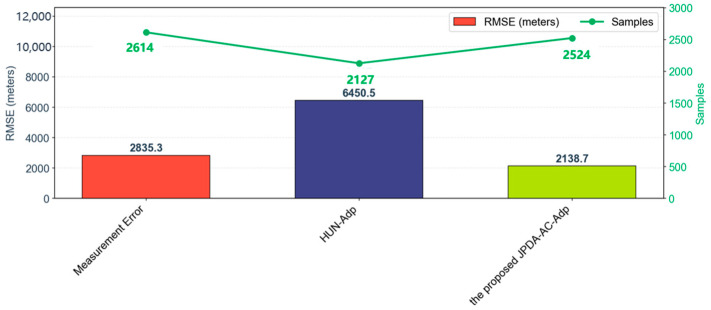
Comparison of tracking position root mean square errors (RMSE) and sample counts among different algorithms under the high-density scenario.

In the dense scenario featuring 30 targets, the baseline RMSE of the raw measurements is 2835.3 m. The statistical error results (Figure 13) indicate that HUN-Adp yields 2127 effective measurement samples with a corresponding tracking RMSE of 6450.5 m, which is more than double the raw observation error. This severe degradation suggests that the Hungarian hard assignment algorithm struggles significantly under high target density, where ambiguity in measurement-to-track association leads to frequent incorrect assignments and track divergence. In contrast, the proposed JPDA-AC-Adp algorithm yields 2524 effective samples with a tracking RMSE of 2138.7 m, successfully maintaining a tracking precision that outperforms the raw observation baseline by a substantial margin, even under exceptionally dense and cluttered conditions.

**Figure 14:**
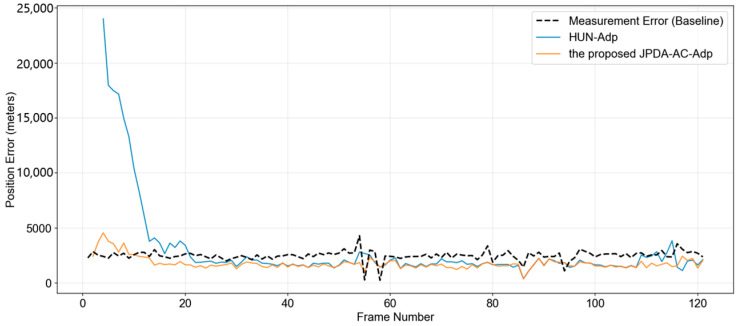
Comparison of frame-by-frame average tracking position root mean square errors (RMSE) among different algorithms under the high-density scenario.

The frame-by-frame tracking error curves (Figure 14) reveal that the Hungarian approach (HUN-Adp) suffers from extreme, anomalous error spikes exceeding 10,000 m during the target initialization and dense cross-over phases, indicating that hard assignment is particularly vulnerable to ambiguity in dense scenarios where multiple targets are closely spaced. In sharp contrast, the proposed annealing-clustering approach (JPDA-AC-Adp) rapidly suppresses these error fluctuations, tightly bounding the tracking error within approximately 2000 m during the stable tracking phase, thereby demonstrating superior clutter suppression and robust track maintenance capabilities.

**Figure 15:**
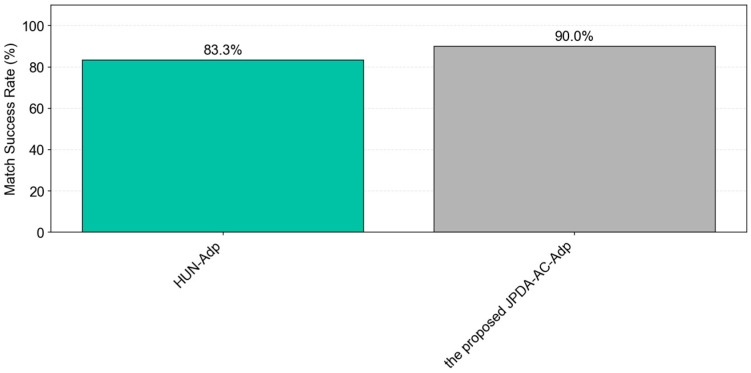
Comparison of track match success rates among different algorithms under the high-density scenario.

**Figure 16:**
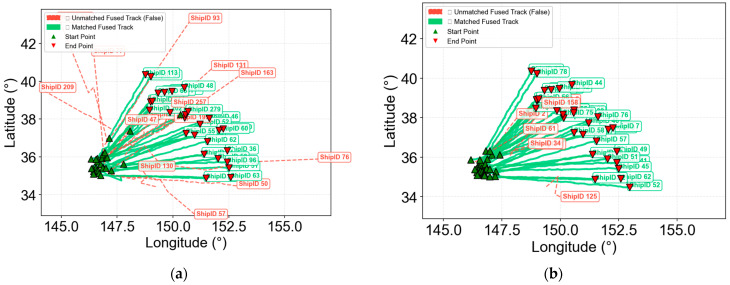
Graphical illustration of the data association results for different algorithms under the high-density tracking scenario: (**a**) HUN-Adp; (**b**) JPDA-AC-Adp.

In terms of trajectory-level track maintenance, the track match success rate (Figure 15) of HUN-Adp drops significantly to only 83.3%, illustrating its vulnerability when handling ultra-dense target intersections. JPDA-AC-Adp achieves a success rate of 90.0%, demonstrating the advantage of soft probabilistic association in dense scenarios. The data association results for the two algorithms are visually compared in Figure 16.

**Figure 17:**
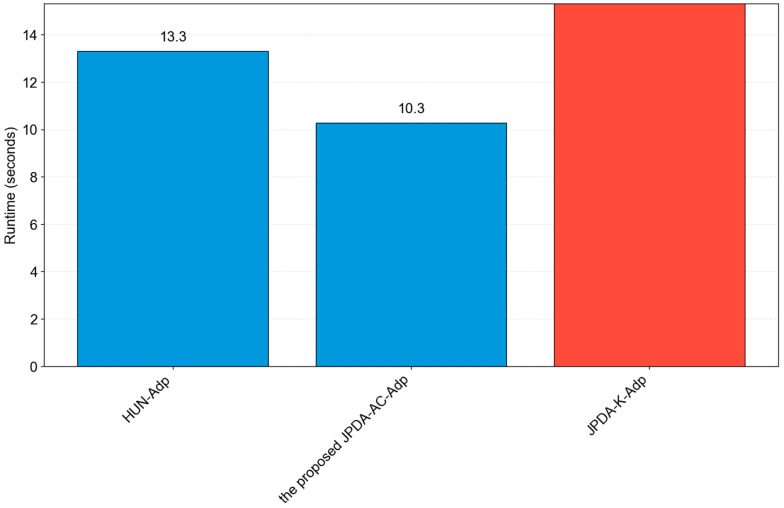
Computational runtime comparison among different algorithms under the high-density tracking scenario (The blue color represents real-time operation, while the red color represents the blocking state of the algorithm.).

The computational runtime profiles (Figure 17) indicate that HUN-Adp requires 13.3 s and JPDA-AC-Adp requires 10.3 s to complete data processing, both fully satisfying real-time execution constraints. However, due to the high target density, the K-best pruning JPDA approach (JPDA-K-Adp) suffers from severe combinatorial explosion, resulting in a computational timeout failure and an inability to return tracking results.

**Figure 18:**
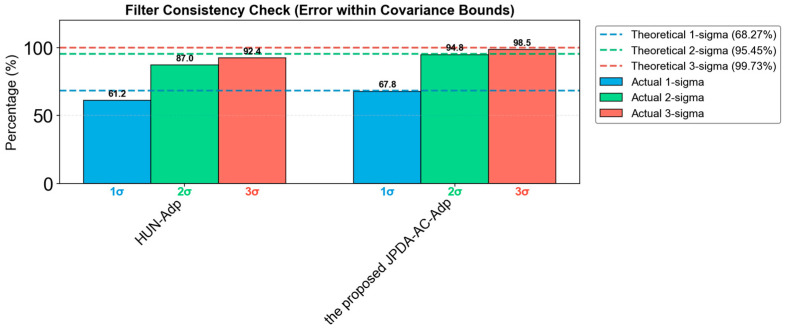
Covariance consistency comparison of different algorithms.

For covariance consistency validation, the theoretical 1σ, 2σ, and 3σ containment rates are 68.27%, 95.45%, and 99.73%, respectively. The proposed JPDA-AC-Adp algorithm achieves a 1 − σ containment rate of 67.8%, deviating from the theoretical value of 68.27% by only 0.47 percentage points, and a 3 − σ containment rate of 98.5%, merely 1.23 percentage points below the theoretical benchmark. These metrics closely approximate the theoretical values across all confidence intervals, demonstrating that the combination of the TIAG mechanism and annealing-based clustering JPDA can precisely maintain the statistical consistency of the covariance estimate. In contrast, HUN-Adp attains a 1 − σ containment rate of only 61.2%, falling 7.07 percentage points short of the theoretical value, and a 3 − σ containment rate of merely 92.4%, a deficit of 7.33 percentage points. Although HUN-Adp also employs the TIAG strategy, its underlying assignment mechanism remains hard decision-making (Hungarian algorithm), which inevitably leads to filter divergence once an incorrect association occurs. The covariance consistency validation results in the dense-scenario experiment independently confirm, from a statistical perspective, that the TIAG mechanism must operate in conjunction with probabilistic soft association to be effective. By virtue of its Bayesian probabilistic association framework, JPDA-AC-Adp performs weighted fusion over multiple candidate association hypotheses, effectively mitigating the adverse impact of any single erroneous association on covariance estimation and thereby safeguarding the statistical consistency of the filter. The covariance consistency comparison is shown in Figure 18.

## 5. Conclusions

To address the critical challenges of discontinuous observations, non-uniform time intervals, sparse spatial measurements, and clutter interference inherent in low-Earth-orbit (LEO) satellite constellation detection for maritime multi-target tracking, this paper proposes a time-adaptive fast data association method tailored for discontinuous and sparse observations. Built upon the theoretical foundation of Joint Probabilistic Data Association (JPDA), the proposed method maintains the probabilistic completeness of the classical framework while introducing systematic innovations across three dimensions: the gating mechanism, the clustering strategy, and the hybrid safety-degradation mechanism.

First, the mismatch mechanism between the Kalman filter predictive covariance and the actual error under discontinuous observations is unveiled. Accordingly, a time-interval adaptive gating (TIAG) mechanism is designed, which dynamically modulates the gating thresholds via a power-law scaling factor, achieving strict conservation of the gating detection probability across arbitrary revisitation intervals and effectively resolving the issues of gating threshold degradation and covariance divergence. Second, to tackle the combinatorial explosion of giant connected components induced by validation matrix densification, a progressive clustering strategy inspired by the simulated annealing philosophy is proposed. This strategy recursively decomposes the global exponential association graph into several subgraphs of manageable sizes, thereby drastically compressing the computational complexity. On this basis, a hybrid safety-degradation mechanism integrating K-best depth-first search heap pruning with Hungarian hard assignment is constructed, guaranteeing numerical robustness and engineering practicality even in extreme scenarios.

Simulation results demonstrate that, compared with conventional fixed-threshold approaches, the proposed innovative algorithm significantly enhances tracking accuracy and track match success rates under varying constellation coverage characteristics and clutter intensities while fully satisfying real-time execution constraints. In conclusion, the method developed in this study provides a high-precision, highly robust, and deployably viable solution for continuous multi-target tracking under discontinuous and sparse space-based observations from LEO constellations.

Despite the encouraging performance demonstrated by the proposed method, several avenues remain open for future investigation. First, while the current framework adopts a constant-velocity (CV) motion model, which is reasonable for open-ocean ships, extending the method to higher-order motion models—such as constant acceleration (CA) or coordinated turn (CT) models—is an important direction for future work, particularly for tracking maneuvering targets in more complex maritime scenarios. Second, the environmental disturbance parameter $\sigma_{a}^{2}$ is currently treated as a pre-specified constant; adaptive estimation of this parameter from online measurements could further enhance robustness under varying sea states. Third, the integration of the proposed method with multi-sensor fusion architectures, including heterogeneous sensors such as AIS and radar, warrants further exploration to improve situational awareness. These extensions will be pursued in our future research to broaden the applicability and robustness of the method.

Furthermore, while the current simulations are based on STK-generated synthetic measurements, applying the proposed method to real AIS data represents a compelling direction for future validation and application. AIS data offers several distinct advantages for this purpose: (1) it provides real-world vessel trajectories with authentic kinematic patterns, including acceleration, turning, and speed variations that are difficult to fully replicate in synthetic scenarios; (2) it naturally exhibits non-uniform temporal sampling due to the varying reporting intervals of different AIS message types and vessel classes; and (3) it allows for large-scale, long-term statistical evaluation across diverse geographic regions and traffic densities. However, several challenges must be addressed when adapting the current method to AIS data. First, AIS messages are typically received by multiple satellites with overlapping coverage, leading to redundant measurements that require careful handling to avoid over-counting. Second, the positional accuracy of AIS data varies significantly depending on the source (e.g., GPS vs. terrestrial navigation systems) and the data transmission format, necessitating robust uncertainty modeling. Third, the ground-truth association for AIS data is not explicitly available; while the Maritime Mobile Service Identity (MMSI) serves as a unique vessel identifier, message loss and corruption may still cause association ambiguities that must be resolved by the tracking algorithm itself. Despite these challenges, the proposed TIAG mechanism and annealing-based clustering strategy are well-suited to AIS data characteristics due to their inherent adaptability to non-uniform sampling intervals and dense target scenarios. Future work will focus on integrating the proposed method with AIS data streams and multi-sensor fusion architectures to further validate its practical effectiveness in real-world maritime surveillance systems.

## Figures and Tables

**Table 1:** Time-interval adaptive gating threshold generation method.

Detailed Steps of the Algorithm
**Input:** Satellite sampling time interval Δt; Baseline gating coefficient $\gamma_{0}$, reference interval $\Delta t_{\mathrm{ref}}$, power-law exponent β; Expansion factor lower and upper bounds $\left[\kappa_{min},\kappa_{max}\right]$. **Output:** Adaptive gating threshold $\theta_{\mathrm{base}}$. **Steps:** : κ←1.0 : **if** Δt≠∅ **and** Adaptive Gating is enabled **then** : $\kappa \leftarrow {\left(\frac{\Delta t}{\Delta t_{\mathrm{ref}}}\right)}^{\beta }▹$ *Power-law expansion model* : $\kappa \leftarrow max(\kappa_{min},min(\kappa ,\kappa_{max}))▹$ *Boundary truncation to prevent overflow* : **end if** : $\theta_{\mathrm{base}}\leftarrow \gamma_{0}\cdot \kappa$ : **return** $\theta_{\mathrm{base}}$

**Table 2:** Construction Method for the Validation Matrix Operator.

Detailed Steps of the Algorithm
**Input:** Track set $T=\{t_{1},\ldots ,t_{N}\}$, Measurement set $D=\{d_{1},\ldots ,d_{M}\}$; Gating threshold $\theta_{\mathrm{base}}$. **Output:** Cost Matrix $C\in R_{\geq 0}^{M\times N}$, Validation Matrix $\Omega \in \{0,1{\}}^{M\times N}$. **Steps:** : κ←1.0 : **if** Δt≠∅ **and** Adaptive Gating is enabled **then** : $\kappa \leftarrow {\left(\frac{\Delta t}{\Delta t_{\mathrm{ref}}}\right)}^{\beta }$ ▹ *Power-law expansion model* : $\kappa \leftarrow max\left(\kappa_{min},min\left(\kappa ,\kappa_{max}\right)\right)$ ▹ *Boundary truncation to prevent overflow* : **end if** : $\theta_{\mathrm{base}}\leftarrow \gamma_{0}\cdot \kappa$ : **for** i=1 **to** M **do** ▹ *Loop through all measurements* : **for** j=1 **to** N **do** ▹ *Loop through all tracks* : $y\leftarrow z_{i}-Hx_{j}$ ▹ *Compute innovation vector* : $S\leftarrow HP_{j}H^{T}+R_{i}$ ▹ *Compute innovation covariance* : **try** : $C_{ij}\leftarrow y^{T}S^{-1}y$ ▹ *Compute Mahalanobis distance* : **end try** : **end for** : **end for** : $\Omega \leftarrow C\leq \theta_{\mathrm{base}}$ ▹ *Generate boolean validation matrix* : **return** C,Ω

**Table 4:** Kinematic parameters of moving targets.

Parameter	Low Density	High Density
Number of Targets	15	30
Time Range	6 July 2025 04:00:00~7 July 2025 04:00:00	
Operational Region	[35° N~36° N, 146° E~147° E]	
Velocity Range (knots)	20~35	
Acceleration Variation Range (m/s^2^)	−0.005~0.005	
Heading Angle Range (deg)	20°~100°	

**Table 5:** Constellation Design Parameters.

Parameter	Configuration A (Sparse)	Configuration B (Baseline)	Configuration C (Dense)
Orbital Altitude (km)	500	500	500
Semi-major Axis (km)	6878.137	6878.137	6878.137
Orbital Inclination (deg)	45	45	45
Number of Orbital Planes (P)	8	10	15
Satellites per Plane (S)	8	10	15
Phasing Factor (F)	2	2	2
Total Number of Satellites	64	100	225
Average Minimum Revisit Interval (s)	174	120	97
Sensor Half-FOV (deg)	45	45	45
Ranging Noise Standard Deviation (km)	2.0	2.0	2.0
Clutter Density (points/m^2^)	1 × 10^−11^	1 × 10^−11^	1 × 10^−11^

## Data Availability

Data are contained within the article.
